# New Frontiers in Stand‐Alone Digital Obesity Treatment

**DOI:** 10.1002/oby.70060

**Published:** 2025-09-28

**Authors:** Delia S. West, Rebecca A. Krukowski

**Affiliations:** ^1^ Department of Exercise Science, Arnold School of Public Health University of South Carolina Columbia South Carolina USA; ^2^ Department of Public Health Sciences, School of Medicine University of Virginia Charlottesville Virginia USA

1

Effective remotely delivered behavioral obesity treatment that does not require direct human interaction holds great promise for achieving the goal of universal access to lifestyle interventions. However, previous research has found attenuated weight loss in digital approaches, particularly among those that do not have a “human touch” [1].

It is in this context that we view the noteworthy work of Thomas and colleagues in reporting the outcomes of their factorial experiment that tested treatment components that all aimed to improve weight loss outcomes for individuals engaged in a digital program [2]. The investigators examined five distinct online components and their combinations using the multiphase optimization strategy (MOST) framework [3] to determine which constellation of these components produced the optimal weight loss outcomes when incorporated into their core digital program. While they found that none of the individual components improved weight loss on its own, the combination of the core online behavioral obesity approach plus interactive video feedback, attention to dysregulated eating, and social support with friendly competition significantly improved weight loss outcomes, with models indicating weight losses of 8.4% at 12 months. The effect of this specific amalgamation of treatment components can be compared with just 3.0% weight loss achieved with the online core treatment alone and 5.9% across the study overall with the various combinations of the treatment components. An 8% weight loss at 12 months produced by a stand‐alone digital intervention without any personnel staffing required could offer a real advancement over existing digital programs; were an effective digital obesity intervention with these outcomes to be broadly disseminated, the potential for positively impacting public health is substantial.

The benefits of utilizing a factorial experiment to optimize digital obesity treatment are highlighted in these results. The effects of the treatment components examined were not additive in the Thomas et al. study [2], likely because engaging in more components can increase participant burden (and perhaps therefore decrease overall engagement) or because different components might potentially achieve the same behavioral goals through the same mechanism and thus be redundant. Indeed, some specific combinations were synergistic, but others were antagonistic (i.e., diminishing the effectiveness of one another). Although the reasons for the antagonistic interaction are not clear, the fact remains that some components were counterproductive when paired together. Further, no significant main effects emerged for any of these treatment components. Thus, part of the value of this factorial study design is in the ability to explore interactions (or combinations of the treatment components added to the basic treatment package). That said, the study was powered to detect significant main effects and markedly underpowered to detect the interaction effects, which is where the beneficial effects were seen.

It is also important to note that the findings of the optimization experiment within the MOST framework are exploratory (for the Thomas et al. study [2] and all optimization phase experiments) and can be compared to a pilot study requiring confirmation. Thus, the Thomas et al. optimized package (i.e., combining the core digital program with video feedback on dietary intake, strategies for coping with emotional eating, and social support with friendly competition) must be tested in a definitive, fully powered trial before firm conclusions about the effectiveness of this package can be drawn and before it should be disseminated broadly.

Perhaps the most profound insight from this study is the need to test combinations of treatment elements to be confident that the components complement and augment one another rather than detract from one another. Within the context of emerging pharmacotherapies for obesity, there are exciting opportunities to pair behavioral strategies with medications to maximize obesity treatment outcomes overall and to begin to tailor treatment packages for the unique individual needs of patients. The current study serves as an example of a “cautionary tale” that combinations of treatment components must not be assumed to be incremental in their benefits or even as simply inert additions. They can also be iatrogenic or have “ill effects.”

In looking forward to the next chapter in obesity treatment in which highly effective medications are available to unite with the behavioral therapy arsenal, it will be important to consider how best to support these medications with adjuvant behavioral components in a way that optimizes overall health while minimizing burden on the individual. With FDA approval for indefinite administration of GLP‐1 and GLP‐1/GIP medications for individuals with comorbid obesity‐exacerbated conditions such as diabetes, the role of behavioral treatment may transition for many individuals into auxiliary supportive therapy to assure healthy weight loss rather than as the primary modality for promoting the weight loss. Preservation of muscle mass, encouraging nourishing, heart‐healthy dietary intake, and promoting sustained adherence to medication prescriptions are likely to become central targets in behavioral treatments supporting the new generation of obesity pharmacotherapies.

To identify optimized treatment combinations in this new era, we will certainly require powerful study designs, such as optimization experiments within the MOST framework, to ensure that we are not taking two steps forward and two steps backward.

Replication of the impressive outcomes of the combined behavioral treatment package suggested by the results of the Thomas et al. study [2] is necessary before moving ahead with extensive implementation of this package, but the potential for a digital program capable of fully autonomous delivery that can achieve outcomes of over 8% weight loss is truly exciting. The Look AHEAD trial demonstrated that weight loss of this magnitude achieved as part of a behavioral lifestyle intervention produced a notable cascade of health improvements across a wide array of disorders [4]. However, the beneficial health outcomes realized in Look AHEAD required an intensive in‐person intervention with highly trained personnel [5], who are not available in all communities. Further, the program was delivered at a cost of over $2800 per participant in the first year of treatment (most of which was staffing costs) [6], which is presumably a much greater cost than was required to implement a year of the optimized digital program in the Thomas et al. investigation, which produced comparable weight loss.

A behavioral obesity treatment that can achieve clinically meaningful weight loss without limitations from geography or personnel accessibility gives us a glimmer of hope that the public health challenges posed by obesity may diminish. With the potential on the horizon for both highly efficacious pharmacological treatments with adjuvant behavioral strategies to maximize overall health and an effective fully digital behavioral alternative for those who do not or cannot access obesity medications, the possibility of a substantive change in the prevalence of obesity and a significant dent in the societal burden associated with the numerous chronic diseases exacerbated by excess adiposity could well be realized in the near future.

## Conflicts of Interest

The authors declare no conflicts of interest.

## Data Availability

Data sharing not applicable to this article as no datasets were generated or analyzed during the current study.
